# Testosterone supplementation and stroke in young adults: a review of the literature

**DOI:** 10.3389/fneur.2024.1422931

**Published:** 2024-09-02

**Authors:** Chetna Dengri, Ahmed Koriesh, Marc A. Babi, Whitney Mayberry, Eric D. Goldstein, Mubashir Pervez, Amre Nouh

**Affiliations:** ^1^Department of Neurology, Cleveland Clinic Florida, Weston, FL, United States; ^2^Department of Neurology, Cleveland Clinic Florida, Port St. Lucie, FL, United States; ^3^Department of Neurology, Brown University, Providence, RI, United States

**Keywords:** stroke in young adults, testosterone, male enhancement drugs, ischemic stroke, hormone replacement therapy

## Abstract

Testosterone supplementation has increased in recent years for both treatment of hypogonadism and recreational use. Strokes in young adults have similarly increased with a larger proportion of patients in this age group having a stroke due to early onset of cardiovascular risk factors or unrelated to conventional risks. Hormonal treatments are associated with increased stroke risk amongst women, with some studies indicating an increase in stroke risk as high as 40% when compared to non-users. However, less is known about male sex hormones and risks associated with increased stroke. Limited data evaluates the relationship between testosterone supplementation and stroke in young adults. In this review, we analyze the literature and plausible underlying pathophysiological mechanisms associated with increased risks in patients using exogenous testosterone. Furthermore, we highlight the gaps in research about safety and long-term effects on young patients.

## Introduction

**Figure 1:**
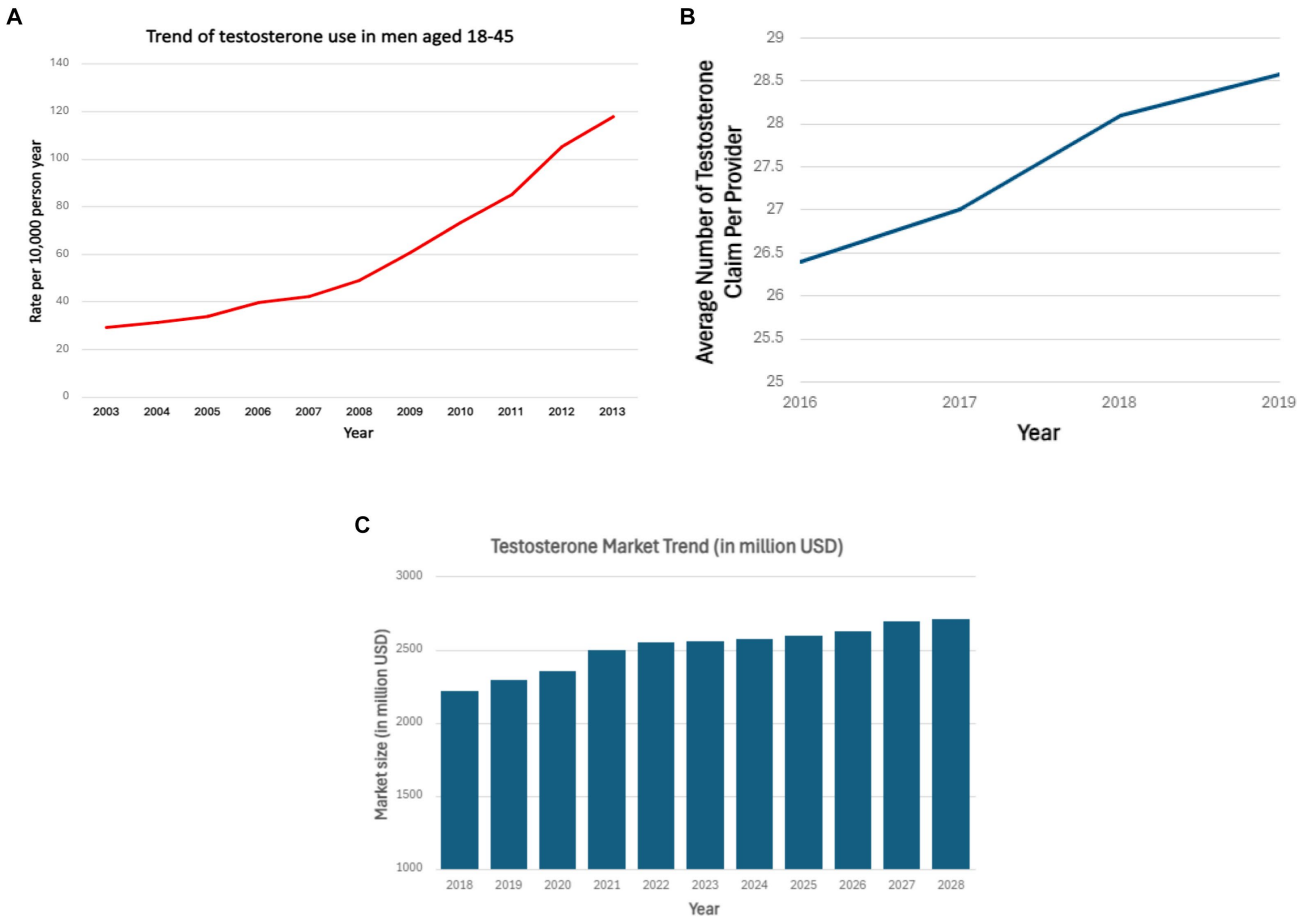
**(A)** Testosterone use among men aged 18–45 years over the period 2003–2013 in the United States (2). **(B)** Average testosterone prescription claims per provider from 2016 to 2019 (6). **(C)** Testosterone market growth in million USD from 2018 to 2028 (7). **(A)** Shows the increasing use of testosterone in men aged 18–45 years from 2003 to 2013 based on outpatient drug claims. Horizontal axis represents years from 2003 to 2013. Vertical axis represents the rate of testosterone use per 10,000-person year. **(B)** Shows increasing average testosterone claim per provider from 2016 to 2018. Horizontal axis represents years from 2016–2019. Vertical axis represents average claim per provider. **(C)** Shows the growth of testosterone market in US, UK, Canada, China, Germany from 2018 to 2028. Horizontal axis represents years from 2018 to 2028. Vertical axis represents market size in million USD.

Testosterone is a steroidal hormone mainly produced by testes in males, and in smaller amounts, by the ovaries or via conversion in adipose tissue in females. As a male sex hormone, it plays a major role in sperm production, libido, muscle, and facial hair production. As illustrated in Figure 1, there has been an increase in the utilization of testosterone supplements across a wide demographic with growth in popularity and public interest in related supplements (1, 2). Hormone replacement therapy involving testosterone is commonly utilized for a broad range of medical indications in addition to off-label applications including recreational consumption in the domain of competitive athletes as well as therapeutic indications for addressing hypogonadism or facilitating gender reassignment procedures (3–5). A study conducted to assess testosterone prescription trends using Medicare prescriber data found an upsurge in the number of prescribers with an annual average increase of 8.8 percent from 2016 to 2019 (6). A recent market analysis forecasts that the testosterone replacement therapy market in the US, UK, Canada, Germany, and China will expand by approximately 500 million USD over the next 4 years (7). However, data on trends of testosterone supplements usage from 2019 to 2024 is currently unavailable. Testosterone supplements are also easily accessible via online stores, limiting accurate quantification of usage rates. While some well-known side effects of testosterone include aggression, acne, coarse facial features, and testicular hypotrophy, other adverse events remain an area of study and debate.

## Background and significance

Previous studies suggested a cardioprotective role of testosterone, as low endogenous testosterone increases cardiovascular morbidity secondary to endothelial dysfunction and increases atherosclerosis (8–10). Conversely, testosterone is known to cause erythrocytosis—leading to possible hyperviscosity and hypercoagulability—as well as a decrease in high-density lipoprotein (HDL), theoretically increasing the risk of cardiovascular disease (11, 12). Prior meta-analyses on this topic have shown conflicting data on the risk of cardiovascular events. These studies were limited to men above the age of 45 years using testosterone for hypogonadism (13, 14). However, little is known about the long-term adverse effects of testosterone supplementation in males without hypogonadism or females taking testosterone supplementation in context of gender assignment or athletic enhancement, given the scarcity of associated randomized trials. Data surrounding prevalence and associated risks, including that of thrombotic events, is even more limited in young adults.

Stroke occurring in patients aged 18–55 years is defined as stroke in young adults (15). Per recent National Health and Nutrition Examination Survey data, there has been an upward trend in the incidence of ischemic stroke in this patient population. In the United States, the incidence of stroke for young adults has increased from 17 per 100,000 in 1993 to 28 per 100,000 in 2015. Stroke in young adults can be attributed to conventional risk factors such as hypertension, hyperlipidemia, diabetes, smoking, obesity, alongside other less common risk factors including hypercoagulability, utilization of estrogen based hormonal supplements, presence of carotid web, vasculopathy, pregnancy/peripartum state, substance use, and migraine with aura (16, 17). While risk factors for stroke in young adults are similar to conventional causes for older patients, thrombotic events occurring in this younger patient population warrant investigation of non-traditional etiologies including thrombophilia and hormonal risks (18). Available literature is most prevalent surrounding stroke in young females taking hormone supplements. This data unequivocally points toward an increased incidence of ischemic stroke in females either taking estrogen only or estrogen-progestin supplements. There is approximately 40 percent higher risk of ischemic stroke in females taking only estrogen-based supplements and around 30 percent risk in females taking estrogen-progestin supplements when compared to placebo (19, 20).

Therefore, despite the increasing prevalence of testosterone and related supplements, the associated implications as a potential vascular risk factor remain underexplored. The lack of data surrounding safety limits physicians’ ability to counsel patients about recreational or therapeutic use with normal baseline endogenous hormone levels. In this review, we aim to summarize the available data and potential implications related to stroke among young adults.

## Methods

A comprehensive literature search was conducted to identify relevant studies on testosterone supplementation and its potential association with stroke in young adults. The databases searched included PubMed Library. The search strategy combined the following keywords and MeSH terms: “testosterone supplementation,” “testosterone replacement therapy,” “stroke,” “cerebrovascular accident,” “young adults,” and “adverse effects.” The search was limited to articles published in English between January 2000 and December 2023.

### Pathophysiological mechanisms of stroke related to testosterone supplementation

Effects of testosterone, whether they are protective or detrimental, are mediated via the androgen receptor and its effect at a cellular level. It is known that exogenous testosterone use induces excitotoxic cellular injury through the glutamate and GABA mediated calcium influx pathway. Stimulation of androgen receptor induced pro-apoptotic p53 target genes. This further leads to cellular inflammation, changes in cell-signaling and damage to blood brain barriers. These findings were demonstrated in animal models of stroke following testosterone use. On the other hand, testosterone imposes its protective effect via the same androgen receptor. Low dose testosterone increases phosphorylation and activity of several factors responsible for neuroprotection (21).

Basic science studies have shown low testosterone levels are associated with low nitric oxide (NO) levels, due to testosterone’s effects on the endothelial nitric NO synthase, phosphodiesterase type 5 (PDE-5), and asymmetric dimethylarginine (ADMA) pathways endothelial progenitor cells (EPC). This can lead to endothelial dysfunction and increased risk of cardiovascular events (22). However, supratherapeutic testosterone levels may also cause harm; research indicates supratherapeutic levels increase oxidative stress and endothelial damage secondary to decreased endothelial NO, mitochondrial reactive oxygen species generation, and NLR family pyrin domain containing 3 inflammasome activation (23, 24).

**Figure 2:**
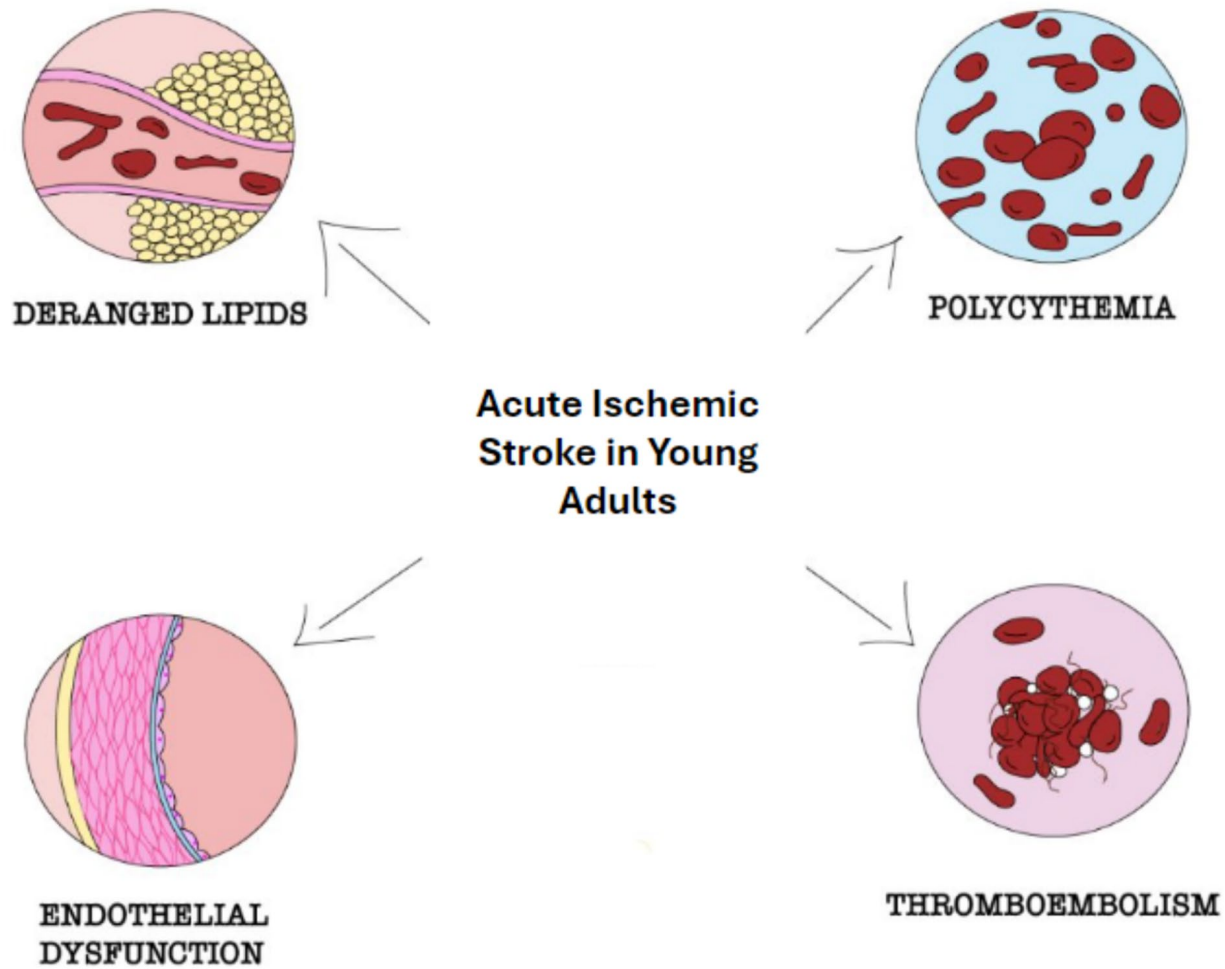
Possible mechanisms of stroke in young adults with supratherapeutic testosterone use. This illustration highlights potential mechanisms of stroke in young adults related to testosterone use.

At the physiological level, endogenous testosterone is known to increase red blood cell mass, increase lean muscle mass, stimulate bone growth, aid in fat redistribution, and decrease low-density lipoprotein (LDL) and triglycerides while increasing HDL. However, at supraphysiological levels, testosterone comes with associated risks. Testosterone is a potent stimulator of erythropoiesis; via multiple mechanisms, it is known to stimulate erythroid colony formation in human bone marrow and stimulate erythropoietin production by kidneys. Thus, supratherapeutic use of testosterone holds a potential side effect of polycythemia causing hyperviscosity of blood, hypercoagulability, and effects on blood pressure (25). Although available data is conflicting, supratherapeutic testosterone is known to cause derangements in plasma lipids through its actions at the cellular level. This includes an increase in LDL and total cholesterol with a concurrent decrease in HDL. Testosterone not only stimulates muscle and bone growth but also increases adipose tissue deposition (26). As a result of these physiological effects, supplementation of testosterone poses the risk of vascular events via increased arterial or venous thromboembolism, hyperviscosity of blood, small vessel disease due to endothelial dysfunction, and derangement of lipid panel (Figure 2).

Limited data is available to study the effects of various forms of testosterone supplements on specified cardiovascular risk factors. Table 1 summarizes the current available data of pathophysiology behind these effects of testosterone on multiple cardiovascular risk factors.

**Table 1:** Literature related to underlying pathophysiological Mechanisms of Stroke Associated with Testosterone Administration.

Associated data by mechanism	Supporting research	Study design	Result
Erythrocytosis	Moriyomo et al., 1975 (25)	*In vitro* research to study the response of bone marrow to increasing doses of testosterone studied *in vitro* versus control.	Statistically significant testosterone dose-dependent increase in erythroid colony formation.
	Maggio et al., 2012 (55)	Sub-analysis of randomized controlled trial (RCT) to study blood samples of 67 men with hypogonadism on transdermal testosterone versus placebo.	Significant increase in hemoglobin (0.86 ± 0.31 g/dL, *p* = 0.01) but no change in erythropoietin (EPO) levels (−0.24 ± 2.16 mIU/mL, *p* = 0.91).
	Bachman et al., 2010 (56)	Sub-group analysis of RCT to evaluate blood factors of healthy young men ages 19–35 and older men ages 59–75 after testosterone supplementation.	Testosterone use was associated with suppression of serum hepcidin. Greater increases in hematocrit were noted in older men related to a greater suppression of hepcidin.
	Bachman et al., 2014 (57)	RCT to evaluate hormonal and hematologic responses to testosterone. Population of men 65 and older with mobility limitations. Randomization to placebo or daily testosterone gel for 6 months.	Increase in hemoglobin and hematocrit after testosterone use was associated with increased EPO and reduced ferritin and hepcidin.
Thromboembolism	Gluek et al., 2018 (58)	Compared patients with venous thromboembolism (VTE) after initiation of testosterone to controls with VTE in the absence of testosterone use.	VTE in patients with testosterone use were more likely to have additional thromboembolic risk factors than controls.
	Walker et al., 2019 (59)	Case crossover study using IBM and Medicare database analysis of 39,622 patients comparing VTE after testosterone therapy in men with and without evidence of hypogonadism.	Testosterone was associated with a higher risk of VTE in men with and without hypogonadism noting an odds ratio of 2.32 (95% CI, 1.97–2.74) and 2.02 (95% CI, 1.47–2.77), respectively.
	Martinez et al., 2016 (49)	Population based case–control study comparing 19,215 patients with confirmed venous thromboembolism with 909,530 age matched controls from source population between January 2001 and May 2013. Further divided into three mutually exclusive testosterone exposure groups were identified: current treatment (>6mon, < 6mon), recent (but not current) treatment, and no treatment in the previous 2 years. Included all men aged 20–89 years with or without hypogonadism.	Adjusted rate ratio of venous thromboembolism was 1.25 (95% confidence interval 0.94 to 1.66) for current versus no testosterone treatment. First 6 months of testosterone treatment, the rate ratio of venous thromboembolism was 1.63 (1.12–2.37), followed by 1.00 (0.68–1.47) after 6 months of treatment. Rate ratio was 1.52 (0.94–2.46) for patients with hypogonadism and 1.88 (1.02–3.45) for those without it.
	Baillargeon et al., 2015 (60)	Case–control study of 30,572 men 40 years and older between January 1, 2007, and December 31, 2012. Cases were defined as men who had a primary diagnosis of VTE and received an anticoagulant drug in the 60 days after their diagnoses. Cases were matched with 3 controls.	Exposure to testosterone therapy in the 15 days before the event/index date was not associated with an increased risk of VTE (aOR, 0.90; 95% CI, 0.73–1.12).
Lipid panel	Agledahl et al., 2008 (12)	Population-based study of total testosterone, sex hormone binding globulin (SHBG), triglycerides (TG), total cholesterol and high-density lipoprotein (HDL) was analyzed in 1274 men without known cardiovascular disease.	A proportional increase in serum TG levels was found in men with total testosterone levels below the 50th percentile. Higher serum testosterone levels were not associated with a change in TG. Total testosterone and SHBG were inversely and independently associated with TGs and positively and independently associated with HDL. Men with HDL less than 0.90 mmol/L and TG greater than 1.80 mmol/L had lower levels of total testosterone and SHBG when adjusted for age and BMI as compared to men with normal HDL and TG.
	Whitsel et al., 2001 (61)	Meta-analysis compared post-treatment lipid profile to pretreatment levels in 272 hypogonadal men.	Treatment with testosterone resulted in post-treatment decreases in HDL, total cholesterol, and LDL for hypogonadal men.
	Isidori et al., 2005 (62)	Systematic review of RCTs evaluating the effect of testosterone administration on serum lipid profile amongst 1,083 participants.	Testosterone use reduced total cholesterol by 0.23 mmol/L (CI: −0.37 to-0.10). Results were most notable in men with lower baseline testosterone concentrations. There was no change in LDL. A significant reduction of HDL cholesterol was found in studies with higher baseline mean testosterone values.

This table summarizes Underlying mechanisms of stroke after testosterone supplementation as identified in existing research.

### Stroke in young adults and testosterone use

Stroke in young adults can not only have a devastating impact on individuals and family but additionally have economic impact. Disability due to stroke can lead to significant costs and productivity losses, especially in young populations. Several studies highlight the economic impact of stroke, however limited data is available to highlight the impact to adults in the working age group. Years of Life Lost (YLL) measures the number of years of life lost due to premature death while Quality-Adjusted Life Years (QALY) is a measure of disease burden and accounts for years of healthy life lived. It is known that younger adults with stroke experienced 4.58 YLL and 9.21 QALYs. Identifying the cause and risk factor of these stroke events in young adults could significantly decrease the stroke burden by lowering the incidence and preventing recurrence (27).

The majority of these strokes are ischemic, and about one-fourth of those fall under cryptogenic stroke with most categorized as embolic strokes of unknown significance (ESUS). In recent years, we have seen a robust increase in over-the-counter exogenous androgen use and incidence of ischemic events (28). As androgens are commonly being utilized without medical advice, their adverse effects—as in erythrocytosis, effects on cardiovascular risk factor, and hypercoagulability potentially leading to ischemic stroke—may go unnoticed and unaddressed in addressing risk factors for decreasing the incidence of stroke in young adults. Most guidelines are published regarding the management of testosterone replacement therapy (TRT) in the setting of hypogonadism. However, there is limited guidance in managing testosterone supplements at a supratherapeutic level (29). Highlighting the incidence of stroke in the setting of testosterone utilization can guide in creating nationwide guidelines for physicians as well as providing education among the general population on adverse events linked to supratherapeutic use of testosterone.

There is known association of aneurysmal subarachnoid hemorrhagic (aSAH) secondary to decreased free testosterone. A randomized mendelian study found that risk of aSAH was higher in postmenopausal females compared to males of the same age group. It is hypothesized that post menopause, there is elevated sex hormone binding globulin (SHBG) that further lowers free testosterone that is associated with increased risk of aSAH. However, further studies are needed in this realm to ascertain the association of supplemental testosterone and incidence of hemorrhagic stroke (30).

Use of testosterone for gender affirmation hormone therapy for female to male transition has been on the rise. Although limited, studies of the young population taking testosterone for gender affirmation have shown statistically significant effects on metabolic parameters. Jarin et al. conducted a retrospective analysis of adolescents taking testosterone or estrogen for gender transition. Seventy two subjects taking testosterone for female to male transition were followed monthly for 6 months showed significant uptrend in hemoglobin/hematocrit levels and BMI, as well as a decrease in HDL (31). Segueira et al., conducted a retrospective chart review to assess the effect on BMI following testosterone use in transmasculine male, that revealed statistically significant uptrend in BMI in the first 6 months after initiation of hormone therapy (32). Gulanki et al. studied the effects of testosterone administration on endothelial function and concluded that androgen use in transgender male and androgen excess in cisgender female was associated with significant decrease in flow mediated vasodilation indicating endothelial dysfunction (33). However, Kennedy et al. conducted a prospective longitudinal study on 59 transgender youth receiving hormone therapy and no significant change in metabolic parameters was noted (34). Table 2 summarizes the available data on effects of testosterone replacement therapy in young adults. However, it is worth noting that these studies were conducted in a controlled setting with regular follow up. Data is scarce that shows the effects of testosterone supplements in young adults who take over the counter testosterone with absence of guided dose or follow up. Further prospective longitudinal studies are warranted to assess the effects of gender affirming testosterone therapy on metabolic parameters.

**Table 2:** Literature related to effects of supratherapeutic testosterone use on cardiovascular risk factors.

Author and year	Study design and age group	Results
Bhasin S. et al., 1996 (63)	Prospective blinded randomized study comparing use of supraphysiologic doses of testosterone with placebo in young athletes, with and without controlled exercise. Ages 19–40.	No effect on lipid profile and hemoglobin/hematocrit. Increases observed in total body weight with testosterone use with or without exercise. Changes in percentage of body fat were not demonstrated.
Karunasena et al., 2016 (64)	Cross validation study surrounding the association of androgens with hemoglobin and hematocrit levels in women with congenital adrenal hyperplasia treated with glucocorticoids. All ages. Mean age 35.3 with SD ± 14.	Higher testosterone levels were noted in women in the highest tertile of hemoglobin and hematocrit levels.
Gulanski et al., 2019 (33)	Direct comparison of endothelial function via flow-mediated vasodilation of 11 transgender men receiving testosterone and 20 cisgender females during the early follicular phase of their menstrual cycle. Transgender males: 27 ± 5. Cisgender females: 28 ± 5.	Statistically significant impairment in vasodilatory response in transgender men on testosterone versus cisgender females.
Sequiere et al., 2019 (32)	Retrospective chart review which collected anthropomorphic data on transmasculine adolescents before and during testosterone use. Ages 13–19.	BMI z-score was significantly higher after 6 months of testosterone use but no significant change was observed between baseline and 12 months. Testosterone dose rate was a significant predictor of change in BMI z-score.
Jarin et al., 2017 (31)	Retrospective analysis of blood pressure, BMI, testosterone, estradiol, prolactin, lipids, electrolytes, liver function tests, hemoglobin, hematocrit, and hemoglobin A1c in affirmed male (female-to-male) patients taking testosterone. Ages 14–25.	Decrease in HDL. Increase in LDL and TG. Increase noted in hematocrit/hemoglobin and BMI.
Kennedy et al., 2018 (34)	Prospective, longitudinal study initially enrolled 101 youth on testosterone therapy for 2 years. Ages 12–23.	Decrease in HDL. Increase in LDL and TG. Decrease in hematocrit/hemoglobin

This table summarizes existing research on implication of supratherapeutic testosterone supplementation on various cardiovascular risk factors.

### Association between stroke subtype and testosterone supplements

Most available studies that investigate an association between TRT and stroke risk use a composite metric for stroke, limiting their interpretation given the heterogeneity of stroke etiologies and pathologies (35–37). In an effort to best understand the impact of TRT on stroke, we identified seven publications which provided delineation of a stroke subtype as ischemic. Overall, there is ambiguity of the impact of TRT for patients with subsequent ischemic stroke. Two studies identified an increased risk while the remaining noted a reduced risk. Six of these studies were retrospective and as such, inherent limitations such as lack of knowledge of medication compliance or robust knowledge of duration of TRT impact their meaningfulness.

Three studies, Vigen et al. (35), Shores et al. (36), and Sharma et al. (38), performed a retrospective cohort study with Veterans Administration electronic medical records from different but overlapping time frames: 2005–2011, 1999–2014, and 2002–2011, respectively. All three studies included only male patients with low serum total testosterone levels receiving transdermal injectable formulations. In general, coexistent hypertension, dyslipidemia, and diabetes mellitus were comparable between the TRT exposed and unexposed. The inclusion criterion was similar amongst the cohort except for Vigen et al. only including patients who underwent a coronary catheterization prior to TRT, and Shores et al. including only men ≥40 years old. The mean follow-up period ranged between 1.5 years and 4.3 years. Vigen et al. identified 33 (2.7%) versus 486 (6.5%) ischemic stroke events and Shores et al. found 383 (4.6%) versus 6,022 (4.9%) ischemic events during the follow up periods. Sharma et al. notes 43 per 100,000 person-years versus 59 per 100,000 person-years ischemic stroke events. In the univariate Cox regression model by Sharma et al., the hazard ratio (HR) was 0.57 (95%CI 0.40–0.82, *p* = 0.002) with TRT unexposed serving as the reference. Additionally, the authors provide a propensity matched HR of 0.64 (95%CI 0.42–0.96, *p* = 0.031) to address potential intergroup biases. Interestingly, this lowered risk was only present in TRT exposed patients who had a normalized serum testosterone level on follow up testing with univariate HR 0.90 (95%CI 0.61–1.34, *p* = 0.61) and propensity matched HR 0.94 (95%CI 0.61–1.44, *p* = 0.68) with unexposed being the reference group helping to address either medication nonadherence or personalized metabolic differences. Shores provides inferential statistical analysis in the context of route of administration of TRT: transdermal delivery unadjusted HR of 0.84 (95%CI 0.69–1.01) for current TRT use, and HR 1.11 (95%CI1.02–1.22) for unexposed with former TRT exposed patients as the reference group, intramuscular delivery unadjusted HR 0.80 (95%CI 0.69–0.93) for active TRT exposure and HR 1.03 (95%CI 0.94–1.13) for unexposed patients referenced to formerly TRT exposed patients. These retrospective cohort studies for males with low baseline serum total testosterone levels treated with testosterone offer differing conclusions. Vigen et al. report an increased risk of ischemic stroke while Sharma et al. conclude normalization of testosterone levels with treatment provided a protective effect against ischemic stroke, and Shores et al. demonstrate no significant risk of ischemic stroke.

Cheetham et al. provides a retrospective Californian cohort study of men ≥40 years old with pre-requisite low blood testosterone (median at entry 212 ng/dL, interquartile range 160–253 ng/dL). The authors use a definition of “ever prescribed” testosterone to capture TRT exposure between 1999 and 2010. In total, they captured 8,808 men in the exposed and 35,527 in the unexposed cohorts with similar intergroup vascular risk factors. A total of 95 and 501 ischemic stroke events occurred for the exposed and unexposed cohorts, respectively, over a mean of 4.4 years. Ischemic stroke event was defined as combined ischemic stroke of transient ischemic attack by ICD coding. This led to 2.1 and 3.1 events per 1,000 person-years for the exposed and unexposed and a hazard ratio of 0.64 (95%CI 0.52–0.80, *p* < 0.001) both after propensity matching by inverse probability of treatment weighting (39). Loo et al. used a similar definition for ischemic stroke, combining ischemic stroke and transient ischemic attack. This study was a retrospective analysis of the Clinical Practice Research Datalink which contained, at the time of publication, over 15 million patient records from 700 primary care practices in the United Kingdom. Patients included in this study were all men aged ≥45 years with documented low testosterone between January 1995 and August 2017, with TRT utilization defined as transdermal or intramuscular use within 90 days of cohort entry. The overall cohort size was 2,237 for the TRT exposed and 13,164 for the unexposed. Over the course of an average of 4.7 years, the authors found 78 ischemic events in the exposed over 9,611 person-years compared with 440 events over 62,043 person-years in the unexposed with an adjusted hazard ratio of 1.23 (95%CI 0.96–1.57) (40).

In the only randomized, multicenter, double-blind, placebo-controlled, parallel group study describing stroke subtype, Brock et al. enrolled males >18 years old with two total testosterone levels documented as low (mean 202.6 ± 66.3 ng/dL) along with symptoms of low-testosterone leading to a sample size of 302 in the interventional arm and 294 in the placebo arm. Participants were provided injectable TRT and followed over 16 weeks. Comparative descriptive data was not provided. The authors report that no participants in the exposed group and one participant in the unexposed group developed an acute ischemic stroke during the 16-week study period. Due to the low event rate, further inferential statistics could not be completed (41).

Finally, Yarnell et al. published a Canadian retrospective cohort study of self-matched patients with prescription data provided by the Ontario Narcotics Registry between October 2012 and October 2017. Unique to this study was that 21 biologically female patients were included, though were unlikely to provide statistical weight given an overall cohort size of 64,386. This study focused on mortality and morbidity secondary to violence after testosterone use. However, the authors reported a relative risk of 1.48 (95%CI 1.25–1.74) of thromboembolic events for patients exposed to TRT when compared to those unexposed for a period of 6 years prior to and 6 years following prescription of testosterone (42). Table 3 summarizes the above stated studies.

**Table 3:** Literature related to cardiovascular events following testosterone supplementation in hypogonadism use.

Author and year of publication	Study design	Inclusion criteria and sample size	Baseline testosterone prior to TRT	Results
Vigen et al., 2013 (35)	Retrospective cohort study	Male undergoing coronary catheterization with hypogonadism. 1,223 case receiving TRT vs. 7,486 control not on TRT	175.5 ng/dL	All-cause mortality, MI, and ischemic stroke Hazard ratio [HR], 1.29; 95% CI, 1.05–1.58 (*p* = 0.02)
Sharma et al., 2015 (38)	Retrospective cohort study	83,010 male with hypogonadism receiving TRT. Divided into 3 groups: 43931 men with Normal T levels after TRT; 25,701 men with Low T levels after TRT, 13378 no TRT.	Unknown	All-cause mortality, MI, and ischemic stroke Hazard ratio (HR): 0.44, confidence interval (CI) 0.42–0.46 were significantly lower in Gp1 vs. Gp3 in propensity-matched cohorts. All-cause mortality, MI, and ischemic stroke HR: 0.53, CI 0.50–0.55 were significantly lower in Gp1 vs. Gp2 (*n* = 25,701, median age = 66 years, mean follow-up = 4.6 years). No difference in MI or stroke risk between Gp2 and Gp3.
Shores et al., 2021 (36)	Retrospective cohort study	204,857 male ≥40 years with hypogonadism. Divided into 3 groups: 122302 with no TRT, 43502 with transdermal TRT, 39053 with intramuscular TRT	220.85 ng/dL	Transdermal testosterone was not associated with risk for the composite cardiovascular outcome (hazard ratio [HR], 0.89; 95% CI, 0.76–1.05) in those without prevalent cardiovascular disease, and in those with prevalent cardiovascular disease was associated with lower risk (HR, 0.80; 95% CI, 0.70–0.91). Current use of intramuscular testosterone was not associated with risk for the composite cardiovascular outcome in men without or with prevalent cardiovascular disease (HR, 0.91; 95% CI, 0.80–1.04; HR, 0.98; 95% CI, 0.89–1.09, respectively).
Cheetham et al., 2017 (39)	Retrospective cohort study	Men aged ≥40 years with hypogonadism. Divided into two groups: 8808 men dispensed TRT and 35,527 never dispensed TRT	Median of 212 ng/dL	Adjusted hazard ratio (HR) for the composite cardiovascular end point in the ever-TRT group was 0.67 (95% CI, 0.62–0.73)
Brock et al., 2016 (41)	Randomized, multicenter, double-blind, placebo controlled, parallel group study	Males >18 years with two testosterone levels <300 ng/dL and 1 symptom of low testosterone. Divided into two groups: 302 on TRT vs. 294 on placebo	202.6 (66.3) ng/dL	No major adverse cardiovascular or venous thrombotic events were reported in the testosterone group. Incidence of increased hematocrit was higher with testosterone vs. placebo (*p* = 0.04).
Yarnell et al., 2021 (42)	Self-matching design	64,386 aged patients >19 years who were treated with TRT; 21 were female	Unknown	**Ischemic stroke events: 410, RR 1.95 (1.56–2.44 95%CI) comparing pre-to post-prescription
Loo et al., 2019 (40)	Cohort of a large registry, Clinical Practice Research Datalink	15,401 men aged >45 years with hypogonadism	Unknown	All-cause mortality was significantly lower with current TRT use (HR 0.64; 95% CI, 0.52–0.78) and higher with past TRT use (HR 1.72; 95% CI, 1.21–2.45), compared with nonuse

This table summarizes existing research on cardiovascular event following testosterone supplementation in men with hypogonadism.

## Discussion

Testosterone supplementation has been touted as an effective enhancer of several key functions, including physical and sexual enhancement. Although large datasets are scarce, meta-analyses found beneficial effects including increased muscle mass, strength, and sexual function (43, 44). These analyses did not demonstrate beneficial effects in cognition or the prevention of cognitive decline (45). Further data from the testosterone trials outlined additional positive effects of supplementation in patients with low testosterone including improvements in all aspects of sexual function, walking, mood and depressive symptoms, hemoglobin concentration in those with mild to moderate anemia, and increased bone density and strength; there was no improvement in vitality. Regarding atherosclerotic risk, a noted increase in coronary plaque volume was present but without an associated increase in cardiovascular events. Ultimately, the duration of treatment was determined to be insufficient, preventing definitive conclusions (46, 47).

Alongside the benefits founds in early data, a small number of trials suggested a potential increase in risk of cardiovascular events and venous embolism—ultimately prompting the Food and Drug Administration to require manufacturers to include labeling warning of an increase in thromboembolism, unrelated to erythrocytosis (48). A couple notable limitations were the observational nature of the study and risk of confounding variables that may affect outcomes as well as time bias. Subsequently, a large meta-analysis including six RCTs and five observational studies concluded that no clear association between VTE and testosterone supplementation existed (49). Therefore, it has been suggested that VTE events related to testosterone supplementation were associated with an underlying and undiagnosed thrombophilia-hyperfibrinolysis (50). This may be of importance in men on testosterone supplementation with history of thrombophilia and mechanism for paradoxical embolism such as a patent foramen ovale or, less commonly, pulmonary arteriovenous shunt.

Out of the many case–control observational studies and few RCTS on testosterone supplementation, heterogeneity regarding adverse events and underlying risk factors has hindered any conclusive data regarding cardiovascular risk. To address this, the TRAVERSE trial (Testosterone Replacement Therapy for Assessment of Long-term Vascular Events and Efficacy Response in Hypogonadal Men) was conducted. This large, randomized placebo-controlled, non-inferiority design trial enrolled men with symptoms of hypogonadism with preexisting cardiovascular risk factors. Both primary and secondary endpoints included non-fatal stroke (as well as non-fatal MI), and tertiary endpoints included death from VTE. Remarkably, there was no difference in primary or secondary endpoints and the majority of tertiary endpoints. However, in the testosterone treatment group, a higher rate of investigator reported adverse events was noted, including non-fatal arrhythmia requiring intervention, atrial fibrillation, pulmonary embolism, and acute kidney injury. This trial provided significant insights to the risks associated with testosterone supplementation (TS) given its robust design and placebo-controlled nature with primary and secondary endpoints including non-fatal stroke with indication that TS over a 22-month course is safe in terms of nonfatal stroke in high-risk individuals, albeit a modest increase in serum testosterone was observed in this study population (37). A meta-analysis of 29 randomized control trials compared placebo and exogenous testosterone use, and found no increase in cardiovascular mortality or morbidity (51).

Testosterone boosters, typically sold as “male enhancement drugs,” have seen a surge in use in recent years with little to no regulation from the FDA (52, 53). These medications often marketed as “natural” or “proprietary blend” with the intent of increasing endogenous testosterone (sometimes as a safe or natural way) include herbal preparations, elemental supplements, amino acids, and vitamins. While data regarding the effect on increasing total serum testosterone is limited, even more limited is the safety profile and relationship to cardiovascular or cerebrovascular events. A recent meta-analysis sought to evaluate the effect of testosterone boosting agents on total serum testosterone. The study evaluated 28 different boosters (with a heterogenous group of active ingredients) including 52 studies with four different patient populations—male athletes, men with late-onset hypogonadism, infertile men, and healthy men—and found the majority of boosters to be ineffective with very few exceptions. Furthermore, only eight studies reported on safety with rare to minimal side effects with only cholecalciferol leading to hypercalcemia in less than 10 percent of patients after 6 months of treatment (54). The attribution of any potential risk may be difficult to discern with boosters in relation to the actual increase in serum testosterone as the risk, or composition of the active ingredients that may include a multitude of substances that are poorly studied or understood. High quality data regarding their utilization and cerebrovascular disease are absent. Of note, the use of anabolic androgens has also risen in athletes, often alongside testosterone use. The use of anabolic steroids has been demonstrated to adversely affect clotting factors, theoretically increasing risk of ischemic stroke. For the purposes of this paper, only research related to testosterone supplementation and related boosters was examined. Lastly, real-world experience is far from related to the effects in RTCs. Inappropriate dosing, treatment for symptomatology rather than truly hypogonadism, and use in younger adults for seemingly perpetuity is a separate setting than strict RCT criteria and provides a unique challenge.

## Summary and future direction

In this review we conclude, based on the best literature available about risks related to a potential stroke mechanism, that there is insufficient data to empirically rule out TS use in the appropriate clinical setting. In some unique patient populations, such as patients with underlying thrombophilia and mechanism for paradoxical emboli, caution and counseling is prudent. In patients who have suffered a stroke, it is important to evaluate the underlying mechanism and exercise judgment in secondary prevention depending on underlying mechanism and stroke subtype. Investigation for thrombophilia and paradoxical emboli is warranted in the appropriate clinical setting. If identified, or there remains high clinical suspicion, consideration of testosterone discontinuation may be warranted.

Further research surrounding the impact of varying formulations on adverse cardiovascular events is warranted. Available data is inclusive of different formulations, dosages, and delivery mechanisms without providing comparative descriptive data. Similarly, research presently suggests differing risks for males with low testosterone versus individuals with normal testosterone levels and continued investigation is necessary. The quality of presently available data remains low, limiting conclusive findings. Overall, as compared to hormonal replacement therapy for women, the associated risks seem low; however, high-quality longitudinal data is lacking and long-term utilization is common among men with symptoms of hypogonadism, with a clear knowledge gap in effects in younger adults with longitudinal use.

It is imperative to exercise caution and adhere to proper protocols in TRT administration to mitigate potential hazards, particularly among populations at elevated risk. Subsequent investigations should emphasize the undertaking of expansive and diverse studies aimed at enhancing comprehension regarding the impacts of TRT on diverse health parameters such as cardiovascular health, prostate cancer, and sleep apnea, encompassing cohorts both with and without predispositions to cardiovascular complications.
